# Possible Association between Obesity and *Clostridium difficile* Infection

**DOI:** 10.3201/eid1911.130618

**Published:** 2013-11

**Authors:** Jason Leung, Bob Burke, Dale Ford, Gail Garvin, Cathy Korn, Carol Sulis, Nahid Bhadelia

**Affiliations:** University of Michigan Hospital, Ann Arbor, Michigan, USA (Leung, J.);; Boston Medical Center, Boston (B. Burke, D. Ford, G. Garvin, C. Korn, C. Sulis, N. Bhadelia)

**Keywords:** Clostridium difficile, obesity, health care-associated infections, inflammatory bowel disease, intestinal microbiome, gut microbiome, bacteria, nosocomial

Inflammatory bowel disease (IBD) is a risk factor for *Clostridium difficile* infections (CDIs). Because of similar disruptions to the intestinal microbiome found in IBD and in obesity, we conducted a retrospective study to clarify the role of obesity in CDI. We reviewed records of patients with laboratory-confirmed CDIs in a tertiary care medical center over a 6-month period. Of 132 patients, 43% had community onset, 30% had health care facility onset, and 23% had community onset after exposure to a health care facility. Patients with community onset had higher body mass indices than the general population and those with community onset after exposure to a health care facility, had higher rates of IBD, and lower prior antibacterial drug exposure than patients who had CDI onset in a health care facility. Obesity may be associated with CDI, independent of antibacterial drug or health care exposures.

*Clostridium difficile* infections (CDIs) have a profound economic effect on the health care system; estimated costs range from $496 million to >$1 billion (*1*,*2*). *C. difficile* is a leading cause of infectious diarrhea in hospitalized patients: the annual number of diagnoses of CDI on discharge has more than doubled, from ≈139,000 to 336,600 during this decade (*3*). The epidemiology of CDI has also shifted. A greater number of community onset cases have been recorded in traditionally low-risk populations (*4*,*5*), raising the concern for whether there are unidentified risk factors increasing the probability of CDI among this subset of persons. Association of CDI with novel risk factors can contribute to improved clinical surveillance of persons at highest risk for infection in the hospital setting or the community.

Inflammatory bowel disease (IBD) has been identified as an independent risk factor for *C. difficile* colonization and disease; patients with IBD have increased severity of illness and death rates from CDI (*6*,*7*). This relationship appears to be modulated by a dysbiosis of intestinal microbiota (*7*,*8*). Similar to studies of antibacterial drugs and IBD, studies have shown that obesity may be associated with decreased diversity and changes in composition of the intestinal microbiome (*9*–*11*). Given the similarities in derangements of the intestinal microbiome seen secondary to antibacterial drug use, IBD, and obesity, obesity may also predispose persons to CDI.

Before 2010, the Society for Healthcare Epidemiology of America and the Infectious Diseases Society of America guidelines (SHEA-IDSA guidelines) defined CDIs as having community onset (CO) or inpatient health care facility onset (HO). Reflecting the changing epidemiology of CDI, the definition was expanded by the 2010 update of clinical practice guidelines to include an additional category of disease: community-onset health care facility–associated (CO-HCFA) (*12*). This category, CO-HCFA, is defined as onset of disease in CDI patients in the community who had exposure to health care facilities during the previous 4 weeks. We believe the introduction of this category has removed cases from the CO cohort who had recent exposure to health care facilities and may help detect associations between CDI and novel risk factors in patients with few other traditional exposures.

This study aims to identify possible demographic and risk factor differences between patients who develop community onset CDI compared with their HO and CO-HCFA counterparts. In particular, we examine whether obesity is overrepresented in patients with community onset infections who did not have exposure to health care facilities, antibacterial drugs, or the diagnosis of IBD. Furthermore, we examine the health care delivery sites represented among patients with CO-HCFA infections. The identification of these sites will facilitate targeted training and education of staff and improved allocation of infection control resources to decrease future incidence of disease.

## Methods

This study was a retrospective analysis of the infection control databases, microbiology results, and medical records of all patients who had laboratory proven CDI at Boston Medical Center (BMC) that serves as a regional safety net hospital. At the time of the study, the 508-bed academic medical center had a network of 15 community health centers. The study was approved by the BMC Institutional Review Board.

Our institution adopted the 2010 SHEA-IDSA guideline classifications for CDI in November 2011. All CDI cases in adults during November 2011–April 2012 were reviewed. Case-patients were defined as persons who had fecal samples positive for *C. difficile* by using the C. Diff Quik Chek Complete enzyme immunoassay (TechLab, Blacksburg VA, USA) or GeneXpert PCR (Cepheid, Sunnyvale, CA, USA) during the study period. At BMC, only non-formed stools are accepted for microbiological analysis for CDI. Samples are tested by enzyme immunoassay for toxins A and B; if the result is inconclusive or clinical suspicion of disease is high, PCR is used.

By using the former classification, the case-patients with laboratory proven CDI were first categorized as having either community or nosocomial onset disease. Patients were then recategorized by using the new SHEA-IDSA guidelines as having CO, CO-HCFA, or HO disease. The CO category included patients who had symptoms or a positive fecal sample test, and no exposure to health care facilities or associated sites for >30 days before the clinic visit or hospital admission. CO-HCFA case-patients were exposed to health care facilities within the previous 30 days. Case-patients with HO disease had onset of symptoms >48 hours after admission and had positive results for CDI laboratory tests. Patients who had new symptoms and a positive assay and a previous positive test for *C. difficile* >30 days but <8 weeks prior to examination were classified as having recurrent disease. Because of the small sample size of this group, recurrent case-patients were excluded from analysis to facilitate statistical comparison.

An exposure to hemodialysis centers, day surgery, chemotherapy suites, or long-term care facilities was considered an encounter with the health care system. Demographic data extracted from the patient chart included age, sex, race and ethnicity, height, and weight. Factors that have been identified as risk factors for CDI were also documented and included the presence of certain coexisting medical conditions, use of anti-ulcer medications, admission to a hospital intensive care unit, duration of hospital stay, and antibacterial use during the preceding month (*13*). IBD was cataloged separately from other immunocompromising conditions. Obesity was defined as body mass index (BMI) >30, calculated as weight (kg)/height (m)^2^.

Statistical analyses were performed by using SPSS software version 20 (SPSS, Inc., Chicago, IL, USA). Descriptive statistics, including student t-test, 1-way analysis of variance, and χ^2^ statistics, were acquired for the data where appropriate. The proportion of CO case-patients diagnosed with obesity was compared with data gathered during 2011 in Massachusetts: population statistics provided by the US Census Bureau (*14*) and weight classification by BMI data provided by the Centers for Disease Control and Prevention Behavioral Risk Factor Surveillance System (*15*). Age was used as a continuous variable to calculate the means in univariate analysis but categorized as either <65 or >65 years for multivariable regression. All reported p values were 2-sided; results with a p value <0.05 were considered significant.

Three binary regression analyses were performed with either CO and CO-HCFA, CO and HO, or CO-HCFA and HO as outcomes. CO-HCFA was used as the reference category for the first model, while HO served as the reference for the second and third models. A stepwise backward elimination method and likelihood ratios were used to find the best fitted model that also contained clinically relevant variables. All 2-way interaction terms were examined; none were found to have a substantial impact. A p value of 0.10 was used for exclusion from the regression model of nonclinically relevant covariates.

## Results

A total of 137 cases of CDI were identified in patients at BMC during the study period. Five patients had recurrent disease and were excluded, and the remaining 132 cases were analyzed. According to the former definitions of location of onset of CDI, 91 cases were CO and 41 were HO. By using the definitions described in 2010, 35.2% (32/91) of the CO cases were found to be HCFA-CO (Figure). Of these, 62.5% (20/32) had a prior hospital admission as a risk factor, while 28.1% (9/32) were from a long-term care facility. Other risk factors (accounting for those with >1 risk factor) included recent surgery (12.5%), hemodialysis (9.4%), or outpatient chemotherapy (3.4%). Results for patient demographics among the 3 CDI categories are shown in Table 1. Among hospitalized patients from each category (109/132), patients with nosocomial infections had a longer length of stay (p<0.001) and were more likely to have been admitted to an intensive care unit (ICU) (p = 0.002) (Table 1).

**Figure:**
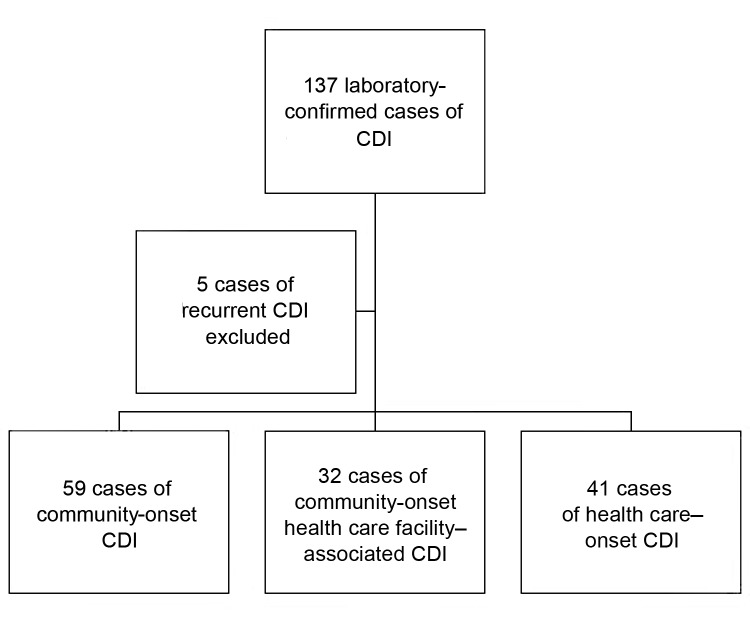
Study population of *Clostridium difficile* infection (CDI) cases.

**Table 1:** Patient characteristics associated with cases of community onset, community-onset health care–associated, and health care onset cases of *Clostridium difficile* infections

Characteristic	Community onset n = 59	Community-onset health care–associated n = 32	Health care onset n = 41	p value
Demographic traits				
Mean age [range]	57.8 [22–96]	63.7 [23–95]	61.6 [21–96]	0.31*
Male sex, no. (%)	27 (46)	10 (31)	23 (56)	0.106
Medication use, no. (%)				
Prior antibiotic use	31 (52.5)	11 (34.4)	33 (80)	<0.001
Antiulcer medication	24 (41)	18 (56)	19 (46)	0.363
Physical status/illness, no. (%)				
Obese (body mass index>30)	20 (34)	4 (13)	13 (32)	0.078
Immunocompromised†	14 (24)	11 (34)	11 (27)	0.551
Diabetes	14 (24)	9 (28)	7 (17)	0.519
Irritable bowel disease	10 (17)	1 (3)	1 (2)	0.018
Required inpatient admission, no. (%)	41 (69)	29 (91)	39 (95)	0.002
Average days stayed	7.7	8.2	18.6	<0.001
Required intensive care unit stay	11 (27)	8 (28)	24 (61)	0.002

*P value for 1-way analysis of variance. †Immunocomprised group included all malignancies, innate and acquired immune conditions such as HIV/AIDS, and congenital immune defects. This group excluded comorbidities listed separately.

In univariate analysis testing for differences across the 3 groups, there were lower percentages of patients with IBD in the HO and HCFA categories compared with the CO group (p = 0.018). A higher percentage of patients in the CO category were noted to be obese, and this finding approached statistical significance (p = 0.08). The percentage of patients in the CO group who were obese (34%) was statistically higher than the state average (23%) (odds ratio 1.7, 95% CI 1.02–2.99). HO cases were more likely to have had prior exposure to antibacterial drugs compared with the CO and HCFA groups (p<0.001). The use of antiulcer medication and coexisting conditions such as immunosuppressive conditions, diabetes, and end stage renal disease were identified with statistically similar frequency in the 3 groups. CDI in HO group was associated with longer hospital stays and higher likelihood of an ICU stay than that in the CO or HCFA groups (p<0.01 for both).

In binomial logistic regression, the CO cohort was noted to be younger (p = 0.03) and 4 times more likely to be obese (p = 0.03) compared with the CO-HCFA group (Table 2). Obesity was not observed at a substantially higher rate in the CO group compared with the CO-HCFA group. The CDIs in CO group were >5 times more likely to be associated with IBD compared with CO-HCFA and ≈6.5 times more likely when compared with the HO group; only the latter comparison approached significance (p = 0.094). Compared with HO patients (p>0.001), CO and CO-HCFA patients were statistically less likely to have had antibacterial drugs before symptom onset (p = 0.01).

**Table 2:** Binary logistic regression analyses of characteristics associated with cases of *Clostridium difficile* infections associated with community onset, community onset–health care-associated, and health care onset

Characteristics	Community onset vs community onset–health care associated*	Community onset vs health care onset*	Community onset–health care associated vs health care onset*	
	Fully adjusted odds ratio (95% CI)			
Age>65	0.35† (0.13–0.92)	0.98 (0.40–2.38)		3.20 (0.91–11.25)
Obesity (BMI>30)	4.06† (1.15–14.36)	1.42 (0.59–3.55)		0.32 (0.07–1.42)
Irritable bowel disease	5.34 (0.61–46.37)	6.40‡ (0.73–56.17)		0.63 (0.03–13.43)
Prior antibiotics	1.91 (0.72–5.11)	0.29† (0.11–0.76)		0.08† (0.02–0.28)
Antiulcer medication	0.62 (0.24–1.63)	0.79 (0.37–1.90)		1.65 (0.52–5.23)

*Reference category. †p<0.05. ‡p<0.10.

## Discussion

This study demonstrates relationships between CDI, IBD, and obesity. By comparing a relatively low-risk group of patients with CDI to those with more traditional risk factors, we sought to identify an association between obesity and CDI. This association was underscored by the hypothesis that in a group without exposure to health care facilities, the statistical significance of other risk factors such as obesity and IBD may be increased. Under the categories created by SHEA-IDSA guidelines, case-patients with CO CDI were 4 times more likely to be obese compared with the community-onset health care facility–associated group, and almost 2 times as likely to be obese as the general population of Massachusetts. Like IBD, obesity may be associated with a higher risk of CDI.

The relationship between IBD and CDI is evolving. Issa et al. (*16*) and Rodemann et al. (*17*) demonstrated that ≈80% of IBD patients who acquired CDI did so in outpatient settings, and another series of inpatients showed IBD patients had CDI onset within an average of 1 day of admission, compared with 4 days for other CDI case-patients. IBD patients received a greater number of antibacterial drugs, had greater exposure to health care facilities, and were frequently administered immunosuppressive drugs that could have increased their risk of infection (*18*). However, there is biologic plausibility that IBD may create an intestinal environment hospitable for CDI, independent of antibacterial drugs and immune modulators.

Studies have demonstrated that the increased incidence of CDI and colonization in IBD patients may be mediated by a derangement of gut flora (*19*). Evolving literature suggests that the community of microorganisms living in symbiosis with the human host affects energy metabolism, alters responses by innate immunity, and can determine outcomes of host pathogen interactions (*20**, **21*). The diversity and the composition of the gut microbial community determine the effectiveness of its symbiosis with the host (*22*). Changes in fecal microbiomes have been demonstrated in recurrent cases of CDI associated with antibacterial drug use (*9*). This defect is also noted in obese patients with IBD (*23*). The similarities in alterations of normal microbial symbiosis in both IBD and obesity may explain why obese patients may be at risk for acquiring CO CDI. Greenblum et al analyzed fecal samples from a cross section of volunteers and examined gene-level and network-level topological differences in intestinal microbiomes associated with obesity and IBD. Obesity and IBD were linked with enzyme level variations and topographical changes, suggesting low diversity environments (*23*).

Aside from the overall decrease in richness of phylotypes of bacterial species, certain host conditions appear to be associated with specific changes in the intestinal microbiota and up or down-regulation of certain bacterial species. The development of CDI appears to be linked to the loss of the ability of the indigenous intestinal species to resist colonization by additional invasive pathogens (*9*). In particular, a decrease in the relative proportion of the phylum Bacteroidetes to that of Firmicutes has been associated with CDI. Manges et al. found that these changes could be driven by antibacterial drugs and health care exposure (*24*). IBD and obesity manifest similar changes in the fecal microbial community (*25*). Obesity may provide a milieu with increased susceptibility for invasion and infection by *C. difficile*.

Higher BMI has been associated with a greater chance by trauma patients of acquiring health care–associated infections, including CDI. In a recent retrospective case control study, Bishara et al. demonstrated a higher BMI in all hospitalized patients with CDI compared with inpatient controls (p<0.001) (*26*). This observation was particularly notable because case-patients and controls had above average BMIs, suggesting that there may be an even more drastic association in the general population. In addition, Bishara et al. noted this relationship between BMI and obesity without differentiation in the probable sites of acquisition (*26*). We were unable to show a difference in obesity between the CO and HO groups, implying that either there is an inherent difference between patients with health care–associated onset and those with hospital onset, or that our study was not statistically powered to detect the association. Because we could show no notable differences between CO-HCFA and HO in regression analysis except for antibacterial drug use, we believe that our study was limited by our sample size.

Use of antiulcer medication has been identified as a risk factor for CDI in the community (*27*). There was no difference in the rate of antiulcer medication use among the 3 subgroups in this cohort. This may reflect local prescribing practices of physicians, because inpatients and outpatients were equally likely to be exposed to these medications. Case-patients who had HO CDI were more likely to have been in an ICU during the study admission. Overall, this trend and the increased incidence of prior antibacterial drug use in this group may represent a higher severity of illness in this cohort. The greater likelihood of nosocomial acquisition of disease could be caused by longer lengths of hospitalization (*28*). Most CO–HCFA case-patients had a history of prior hospitalization. Long-term facilities, day surgery centers, and outpatient hemodialysis sites appear to also serve as potential sites of increased transmission of CDI outside the hospital.

The main limitation of this study is related to the lack of data for true prevalence of risk factors in each group, because we compared cases with each other on the basis of the location of onset and not to controls. Hence, the trends observed require further validation with prospective analysis to establish whether there is a true association between obesity and CDI as noted in the CO cohort. The analysis is also limited by the retrospective design and, as mentioned before, the relatively small sample size. In addition, data collection was dependent on chart extraction, hence dependent on provider documentation. Since cases were defined by patient samples with positive diagnostic assays, this study did not differentiate between patients who were colonized and those with active disease. This may have overestimated true disease prevalence, as has been demonstrated (*29*). However, because only non-formed fecal samples are accepted for analysis at our laboratory, it is likely that the majority of the cases represented true disease.

## Conclusions

Translational research could help elaborate the dimensions of the interaction of the intestinal microbiota with *C. difficile* in obese patients. It would also be of interest to establish if there is a dose response between BMI and risk for CDI acquisition. Further, it is critical to establish whether obesity is a risk factor for high rates of *C. difficile* colonization, as is IBD; if that risk factor is established, prospective observations would improve understanding of whether obesity plays a role in the acquisition of CDI, or alters severity of disease and risk for recurrence. Last, the examination of the CO–HCFA group in this study underscores the importance of increased infection control at ancillary health care facilities and surveillance for targeting high-risk patients who were recently hospitalized.
